# Physical Fighting and Associated Factors among Adolescents Aged 13–15 Years in Six Western Pacific Countries

**DOI:** 10.3390/ijerph14111427

**Published:** 2017-11-21

**Authors:** Lili Yang, Yuanyuan Zhang, Bo Xi, Pascal Bovet

**Affiliations:** 1 Department of Epidemiology, School of Public Health, Shandong University, Jinan 250012, China; yangll221@163.com (L.Y.); yuanyuanzhang2010@hotmail.com (Y.Z.); 2 Institute of Social and Preventive Medicine, University Hospital Center, 1010 Lausanne, Switzerland

**Keywords:** youth violence, physical fighting, smoking, alcohol drinking, adolescents, Western Pacific, low- and middle-income countries

## Abstract

Youth violence is an important public health challenge around the world, yet the literature on this problem in low- and middle-income countries (LMICs) has been limited. The present study aims to examine the prevalence of adolescent physical fighting (defined as having been involved in at least one physical fight during the past 12 months) in selected LMICs, and its relations with potential risk factors. We included 6377 school-going adolescents aged 13–15 years from six Western Pacific (WP) countries that had recently conducted a Global School-based Student Health Survey. Information was gathered through a self-administered anonymous closed-ended questionnaire. The prevalence of adolescent physical fighting varied across countries, ranging from 34.5% in Kiribati to 63.3% in Samoa. The prevalence was higher in boys than in girls, and lower at age 15 than 13–14 years. Physical fighting was significantly associated (pooled odds ratios (ORs), 95% confidence intervals (CIs)) with smoking (1.78, 1.53–2.06), drinking (1.57, 1.33–1.85), drug use (1.72, 1.33–2.23), and missing school (1.72, 1.51–1.95). The association with physical fighting increased with increasing number of joint adverse behaviors (increased from 1.99 (1.73–2.29) for one risk behavior to 4.95 (4.03–6.07) for at least 3 risk behaviors, versus having none of the 4 risk behaviors). The high prevalence of physical fighting and the associations with risk behaviors emphasize the need for comprehensive prevention programs to reduce youth violence and associated risk behaviors.

## 1. Introduction

Youth violence is an important public health issue worldwide, with physical fighting being a common manifestation [1,2,3]. In Europe and North America, adolescent fighting prevalence varies across countries, from 37% to 69% in boys and 13% to 32% in girls [4]. Due to increased public awareness and targeted intervention programs, the prevalence of adolescent fighting has decreased in high-income countries (HICs) [5,6]. According to the 2015 Youth Risk Behavior Survey (YRBS), 22.6% of high school students in the United States were involved in a physical fight within the past 12 months [7]. The prevalence of adolescents participating in physical fighting seems to be high in low- and middle-income countries (LMICs) as well, though few studies are available in these countries, and several of them rely on data collected 10 or more years ago. In Namibia, in 2004, 50.6% of adolescents reported having been engaged in at least one physical fight in the past year [8]. In Egypt, in 2006, 31% of adolescents aged 11–17 years had been engaged in a fight within a 12-month recall period [9]. In Ghana, in 2007, 32% of adolescents had been involved in two or more episodes of physical fights during the last 12 months [10]. In 2012, 27% of Malaysian adolescents reported having been in a physical fight during the last 12 months [11].

Physical violence is a major cause of youth morbidity and mortality, and was ranked the fourth leading cause of death in 2013 among adolescents aged 15–19 years old in the world (5.5%) [12]. It has been shown to be strongly associated with unhealthy behaviors such as alcohol and drug use [8,9,13]. Additionally, youth violence tends to track into adulthood [14,15].

Considering the magnitude and severity of this issue, the World Health Organization (WHO) has called for concerted efforts to prevent physical violence in both youth and adults worldwide and to build global commitments to violence prevention [1,2,3]. However, only one third of the 133 surveyed countries had implemented large-scale initiatives to reduce violence in both youth and adults in 2014 [3]. Healthy People 2020 set targeted objectives to reduce physical fighting among adolescents by 10% in the United States between 2009 and 2020 [16].

In order to better inform youth violence prevention strategies, a growing body of literature has focused on risk or protective factors associated with physical fighting among youth [5,13,17]. However, most of the available evidence comes from developed countries, particularly Europe and North America, and these findings may not necessarily generalize to LMICs. Therefore, we carried out the current study using data from 6 Western Pacific (WP) countries that had conducted a Global School-Based Student Health Survey (GSHS) and had data available on physical violence and selected potential risk factors, including smoking, drinking, drug use, and missing school. Such information can be helpful to guide policy and programs to address physical violence and its associated risk behaviors.

## 2. Methods

### 2.1. GSHS

The GSHS, an ongoing surveillance project, has been developed by the WHO and the US Centers for Disease Control and Prevention. Its goal is to provide data on health and risk behaviors among school-going adolescents aged 13–15 years in LMICs in order to assist these countries in establishing programs and policies accordingly. Details on methods and main results (country fact sheets) of the GSHS are available at http://www.cdc.gov/gshs/.

In each GSHS-participating country, a two-stage probability sampling strategy was used to generate a representative sample of all students aged 13–15 years. In the first stage, schools were selected with a probability proportional to their enrollment size. In the second stage, classes in the selected schools were randomly chosen. All students in these classes were eligible to participate in the survey, irrespective of their actual age. They were invited to complete, in class, a self-administered questionnaire. They provided answers on a computer-scannable answer sheet, which was subsequently added to a database using optical character recognition at the US CDC. The survey was anonymous. The GSHS questionnaire was made of several modules (e.g., HIV, nutrition, tobacco use, etc.), of which countries are free to select when they conduct the survey. However, countries are not allowed to change the wording of the questions, so that results can be compared directly between countries and over time. Two years after a survey is completed, data files are freely available to the public from the web site of the US CDC.

Six countries from WP (Cook Islands, Kiribati, Samoa, Solomon, Tonga, and Vanuatu) had gathered information on physical fighting (module “violence and unintentional injury”) and selected variables of interest (i.e., smoking, drinking, drug use, and missing school without permission, which appear in modules on “tobacco use, alcohol use, drug use and protective factors”). After excluding subjects aged younger than 13 and older than 15, as well as those with missing data on the variables of interest, a total of 6377 (boys: 47.5%) young adolescents were included in the final data analyses.

All subjects and their parents/guardians gave the informed consent for inclusion before they participated in the study. The GSHS database is publically open for use.

### 2.2. Definition of Variables

#### 2.2.1. Physical Fighting

A question on physical fighting appears in the module “violence and unintentional injury”. The question reads: “During the past 12 months, how many times were you involved in a physical fight?” with eight given possible answers, ranging from “0 time”, “1 time”, “2 or 3 times”, “4 or 5 times”, “6 or 7 times”, “8 or 9 times”, “10 or 11 times” to “12 or more times”. 

#### 2.2.2. Smoking

Questions on smoking appear in the module “tobacco use” and questions considered in this study read as follows: “During the past 30 days, on how many days did you smoke cigarettes?” and “During the past 30 days, on how many days did you use any tobacco products other than cigarettes, such as tobacco roll, snuff or chew tobacco?” and possible answers were: “0 day”, “1 or 2 days”, “3 to 5 days”, “6 to 9 days”, “10 to 19 days”, “20 to 29 days” and “all 30 days”.

#### 2.2.3. Drinking

The question on alcohol use considered in this study came from the module “alcohol use” and reads “During the past 30 days, how many days did you have at least one drink containing alcohol?” and possible responses were: “0 day”, “1 or 2 days”, “3 to 5 days”, “6 to 9 days”, “10 to 19 days”, “20 to 29 days” and “all 30 days”.

#### 2.2.4. Drug Use

The questions on drug use came from the module “drug use” and read: “During your life, how many times have you used marijuana (also called weed, grass, joint (or any culturally appropriate word in the country))?” and “During your life, how many times have you used amphetamines or methamphetamines (also called tik, etc. (or any culturally appropriate word in the country))?” Responses were: “0 time”, “1 or 2 times”, “3 to 9 times”, “10 to 19 times” and “20 or more times”.

#### 2.2.5. Missing School

Missing school without permission was assessed with the question, “During the past 30 days, on how many days did you miss classes or school without permission?” with possible responses being: “0 day”, “1 or 2 days”, “3 to 5 days”, “6 to 9 days” and “10 or more days”.

All these variables were recoded as negative (coded 0) for 0 times/days and positive (coded 1) for one or more times or days. Additionally, for the Pearson partial correlation analysis, a continuous measure of every variable was derived by taking the midpoints or values of the responses, e.g., for physical fights, the highest option, “12 or more times”, was top-coded at 12.5 times [18].

### 2.3. Statistical Analysis

Data analysis was performed using SAS software V. 9.3 (SAS, Cary, NC, USA). Analyses were performed using the weighting variables from GSHS corresponding to the two-stage sampling strategy of the GSHS. Pearson partial correlation analyses and logistic regression models were used to estimate the relationship between physical fight and the selected risk factors, i.e., age, sex, smoking, drinking, drug use, and missing school. In the multivariate logistic analysis, each independent variable was regarded as the main exposure, controlling for the other independent variables (including the other risk factors). We also evaluated the joint influence of risk behaviors (smoking, drinking, drug use and missing school) on the risk of physical fighting using multivariate logistic analysis after adjustment for age and sex. The results were presented as odds ratios (OR) and 95% confidence intervals (CIs). Pooled estimates in the WP region were calculated by meta-analysis using Stata 11.0 (StataCorp LD, College Station, TX, USA). *p* values less than 0.05 indicate statistical significance. The Bonferroni correction method was applied for multiple comparisons.

## 3. Results

### 3.1. Sample Characteristics

Characteristics of the country samples are presented in Table 1. A total of 6377 adolescents aged 13–15 years old were included in the data analysis, with boys accounting for 47.5%. The sample sizes ranged from 638 in Vanuatu to 1667 in Tonga. The response rate was lowest in Samoa (67.9%) and highest in the Cook Islands (95.6%).

### 3.2. Prevalence of Physical Fighting, Smoking, Drinking, Drug Use, and Missing School

The pooled prevalence of adolescents engaged in one or more physical fights during the past 12 months was 47.2% (boys: 52.8%, girls: 42.2%) in the WP region. However, the prevalence varied across countries. The highest prevalence was 63.3% in Samoa, and the lowest was 34.5% in Kiribati. In general, physical fight was more prevalent in boys than girls. The pooled prevalence of smoking, drinking, drug use, and missing school were 29.5%, 25.5%, 15.3% and 43.7%, respectively, in boys; the corresponding values were 20.5%, 17.5%, 8.5% and 37.6% in girls (see Table 2).

### 3.3. Risk Factors for Physical Fighting

In the partial correlation analyses, smoking, drinking, drug use and missing school were all positively associated with physical fighting in both genders after controlling for age (see Table 3).

After controlling for the other covariates, the multivariate logistic regression analysis showed that male adolescents and those who reported to have experienced smoking, drinking, drug use or missing school in the past 30 days were more likely to engage in physical fighting than those who did not. The pooled ORs (95% CIs) of smoking, drinking, drug use and missing school were 1.78 (1.53–2.06), 1.57 (1.33–1.85), 1.72 (1.33–2.23), 1.72 (1.51–1.95), respectively. Compared with those aged 13–14 years, adolescents aged 15 years were less likely to engage in fights in pooled analysis, although the difference was not significant at the country level (see Table 4). The statistical significance did not change for most subgroups even after the Bonferroni correction (0.05/42 = 0.0012).

The likelihood for physical fighting increased with increasing number of risk behaviors (smoking, drinking, drug use, and missing school), after adjusting for age and sex. Compared to subjects without risk behaviors, those with 1, 2 and ≥3 risk factors were more likely to engage in physical fighting. The pooled ORs (95% CIs) were 1.99 (1.73–2.29), 3.52 (2.94–4.21), 4.95 (4.03–6.07) (see Table 5). The statistical significance did not change for most subgroups even after the Bonferroni correction (0.05/21 = 0.0024).

## 4. Discussion

The present study shows that adolescent physical fighting is a common problem in the WP region, with a pooled prevalence of 47.2%, which is similar to or higher than in developed and other developing countries [8,9,11,13,17]. The prevalence varied across countries, ranging from 34.5% in Kiribati to 63.3% in Samoa. These differences may be due to different cultural norms and societal tolerance between countries with regards to adolescent physical fighting [5,19]. 

In line with previous studies [5,8,11,19], the prevalence of physical fighting was higher in boys than girls in the WP region. It has been suggested that a gender difference may be due to larger societal tolerance of physical violence among males vs. females [20]. In some societies, physical violence may be interpreted as being part of the male status, while females may be more likely to be verbally aggressive [21]. Biological differences in levels of sexual hormones (e.g., testosterone) may also underlie some differences in levels of physical violence between boys and girls. Boys are also more likely to be engaged in risk behaviors associated with physical violence than girls, such as alcohol drinking or substance use. However, in Solomon and Tonga, the gender difference in physical fighting was not significant, which is consistent with some recent studies showing that the gender gap in risk behaviors may be narrowing over time [22,23]. Narrowing gaps in both physical violence and behavioral risk factors (smoking and substance use) may be related to trends toward greater gender equality, which are also promoted by the industry with regards to some risk behaviors (e.g., campaigns promoting smoking and alcohol targeting at girls), as there is a larger potential for increasing consumption of these substances among girls than boys in many countries. One can also speculate that interpersonal physical violence, which is increasingly being recognized as inadequate, may be shifting to other more insidious and less traceable forms of violence [24].

Some previous studies have shown that the prevalence of physical fighting declined with age among adolescents [17,25]. As fighting may be regarded as a method of solving conflicts for young adolescents, an age-related decline might reflect the development with age of non-violent skills to solve conflicts [17]. Furthermore, parents may be more likely to neglect fighting behaviors of younger adolescents as they tend to be less likely to cause serious physical injuries [17]. In this study, the pooled prevalence of physical fights among those aged 13–14 years was higher than those aged 15 in the WP region, although the difference was not significant at a country level. This may be due to the narrow age range in this study.

Consistent with other studies [8,9,11,13,26], the present study also showed associations between physical fighting and smoking, drinking, drug use and missing school in the 6 considered LMICs in the WP region. Alcohol and drug consumers are often unaware of becoming aggressive, thus promoting the occurrence of violence [27,28]. Research on the relationship between missing school and physical fighting has been limited [29], largely because missing school is often regarded as a product of social pressure or inequality. However, as peer pressure contributes to physical fighting and other risk behaviors such as alcohol use [30], it is reasonable to think that students who have been involved in physical fights are more likely to miss school without permission through a complex, indirect way [10]. Furthermore, the prevalence of risk behaviors associated with physical violence (smoking, alcohol use, substance use) is larger in students missing school than those not missing school [31].

Our study also showed that the physical fighting increased greatly with increasing clustering of risk behaviors. This result supports previous research results suggesting that being engaged in physical fighting could be a useful indicator of multiple-risk-behavior syndrome [32]. Hence, programs targeting at risk behaviors simultaneously and comprehensively are useful to effectively address both the considered risk behaviors and reduce physical violence.

The present study adds to the limited existing literature on adolescent physical fighting in LMICs. Furthermore, population-based sampling and the use of standardized methods and same questionnaire in the GSHS project allow direct comparison of findings across countries. However, there are several limitations to this study. First, the questionnaire was self-administered, and answers may be misreported in unpredictable ways. Since the physical violence and risk behaviors considered are generally not well accepted, socially, especially among girls, responses might be biased towards socially desirable forms. This may result in regression of estimates to the null values, and underestimate the magnitude of the association estimates between physical violence and risk behaviors. Furthermore, participants were required to recall information related to event occurring 12 months prior, which have led to inaccuracy in the responses. Second, the study only included in-school adolescents, who tend to have fewer risk behaviors than students not in school [31,33]. Third, causality cannot be inferred from the cross-sectional design. Fourth, the GSHS questionnaire only assessed the number of physical fights a child had been involved in during the past 12 months, with no further information collected on the severity of the fight or whether the respondent was the victim or the offender. Additional research is needed to examine physical fighting and other forms of violence among youth, including more detailed characterization of fighting patterns, circumstances, causes and impacts.

## 5. Conclusions

In summary, physical fighting among school-going young adolescents aged 13–15 years old was fairly frequent in WP countries, and was associated with several risk behaviors. These findings may be useful to advocate and guide policy makers with regards to implementation of strategies to prevent both unhealthy behaviors and physical violence among adolescents. Schools constitute a particularly suitable setting to address physical fighting and associated risk factors among adolescents, by providing counseling and skills to cope and resolve conflicts, through programs involving teachers, peers and parents. The clustering of physical violence with other risk behaviors emphasizes the need for integrated prevention programs address multiple risk behaviors and physical violence. More generally, a broad range of stakeholders in the society (government, NGOs, media, parents’ associations, etc.) needs to join forces to effectively fight against all forms of violence, including verbal violence, bullying, sexual harassment, and victimization. This includes developing policy and regulations, for schools and other settings, to define what is not acceptable; training teachers, sport trainers, and other key personnel in other sectors to respond adequately to violence cases and to be able to run prevention programs; consistently enforcing strong sanctions against offenders; and, perhaps most importantly, building social norms that do not tolerate violence.

## Figures and Tables

**Table 1:** Characteristics of the Global School-Based Student Health Surveys in 6 low- and middle-income countries in the Western Pacific region.

Country	Survey Year	Response Rate %	Sample Size	Boys %	Age Range
Cook Islands	2011	95.6	711	49.4	13–15
Kiribati	2011	94.3	1217	44.7	13–15
Samoa	2011	67.9	1412	44.9	13–15
Solomon	2011	86.5	732	52.3	13–15
Tonga	2010	91.0	1667	50.1	13–15
Vanuatu	2011	91.4	638	46.7	13–15

**Table 2:** Prevalence of physical fighting, smoking, drinking, drug use, and missing school in young adolescents in 6 Western Pacific countries.

Country	Sex	*N*	Physical Fight		Smoking		Drinking		Drug Use		Missing School	
			%	SE	%	SE	%	SE	%	SE	%	SE
Cook Islands	Boys	351	46.4 **	1.9	20.5	1.5	27.9	1.7	10.8 *	1.2	35.0 *	1.8
	Girls	360	33.3	1.8	19.7	1.5	28.9	1.7	6.4	0.9	25.0	1.6
Kiribati	Boys	499	43.0 **	2.9	39.6 **	3.7	43.1 **	3.3	9.2 **	1.2	37.8 *	3.1
	Girls	718	27.7	2.1	23.2	2.5	19.2	2.1	2.0	0.6	29.9	2.3
Samoa	Boys	538	71.4 **	2.9	49.5 **	3.7	39.4 **	3.5	46.3 **	3.3	58.2 **	3.1
	Girls	874	56.6	2.5	26.7	2.8	22.5	2.2	24.2	2.8	44.6	2.9
Solomon	Boys	369	52.4	4.3	28.9 *	3.7	19.7	4.0	19.4 *	3.7	45.0	5.3
	Girls	363	46.2	5.8	20.5	3.1	12.5	2.3	13.0	2.0	43.6	4.0
Tonga	Boys	744	45.3	2.5	20.3 *	1.9	13.5	1.6	5.9	1.1	36.1	2.3
	Girls	923	48.7	2.1	25.6	2.0	17.1	1.8	8.1	1.2	41.2	1.9
Vanuatu	Boys	250	58.8 *	3.8	19.1 *	4.7	10.3 *	2.6	4.5	1.4	52.8 *	5.4
	Girls	388	41.5	4.6	7.8	1.5	5.2	1.2	2.0	0.6	43.0	4.2
Pooled	Boys	2751	52.8	4.4	29.5	4.4	25.5	5.0	15.3	3.4	43.7	4.0
	Girls	3626	42.2	5.0	20.5	3.3	17.5	4.1	8.5	1.9	37.6	3.9

*p* indicates comparisons between sex in each country; * indicates *p* < 0.05, ** indicates *p* < 0.001; SE = Standardized Error.

**Table 3:** Age-adjusted partial correlation coefficients of physical fighting with smoking, drinking, drug use and missing school in young adolescents in 6 Western Pacific countries.

Country	Boys								Girls							
	Smoking		Drinking		Drug Use		Missing School		Smoking		Drinking		Drug Use		Missing School	
	r	*p*	r	*p*	r	*p*	r	*p*	r	*p*	r	*p*	r	*p*	r	*p*
Cook Islands	0.29	<0.001	0.23	<0.001	0.11	0.040	0.13	0.016	0.22	<0.001	0.21	<0.001	0.12	0.020	0.13	0.016
Kiribati	0.07	0.135	0.03	0.514	0.08	0.084	0.01	0.850	0.03	0.377	0.08	0.024	0.24	<0.001	0.15	<0.001
Samoa	0.24	<0.001	0.17	<0.001	0.23	<0.001	0.14	0.002	0.35	<0.001	0.27	<0.001	0.25	<0.001	0.18	<0.001
Solomon	0.27	<0.001	0.30	<0.001	0.29	<0.001	0.20	<0.001	0.22	<0.001	0.24	<0.001	0.26	<0.001	0.21	<0.001
Tonga	0.18	<0.001	0.13	<0.001	0.17	<0.001	0.18	<0.001	0.25	<0.001	0.23	<0.001	0.23	<0.001	0.15	<0.001
Vanuatu	0.23	<0.001	0.10	0.112	0.24	<0.001	0.16	0.014	0.09	0.086	0.03	0.618	0.01	0.786	0.07	0.185
Pooled	0.21	<0.001	0.16	<0.001	0.18	<0.001	0.13	<0.001	0.20	<0.001	0.18	<0.001	0.19	<0.001	0.15	<0.001

r means age-adjusted partial correlation coefficient.

**Table 4:** Influence of smoking, drinking, drug use and missing school on risk of physical fight in young adolescents in 6 Western Pacific countries.

Country	Age 15 vs. 13–14			Boys vs. Girls			Smoking			Drinking			Drug Use			Missing School		
	OR	95% CI	*p*	OR	95% CI	*p*	OR	95% CI	*p*	OR	95% CI	*p*	OR	95% CI	*p*	OR	95% CI	*p*
Cook Islands	0.54	0.38–0.77	0.001	1.72	1.25–2.37	0.001	1.67	1.08–2.57	0.022	1.62	1.10–2.39	0.016	1.79	1.00–3.22	0.051	1.87	1.32–2.65	<0.001
Kiribati	0.88	0.60–1.29	0.514	1.59	1.24–2.04	<0.001	1.61	1.25–2.08	<0.001	1.47	1.11–1.95	0.008	1.60	0.89–2.87	0.120	1.41	1.13–1.76	0.003
Samoa	1.02	0.78–1.33	0.872	1.40	1.01–1.95	0.044	2.01	1.45–2.79	<0.001	2.15	1.52–3.03	<0.001	1.18	0.73–1.89	0.499	1.62	1.11–2.36	0.012
Solomon	0.82	0.53–1.28	0.380	1.20	0.70–2.04	0.508	1.02	0.58–1.78	0.956	1.38	0.68–2.81	0.368	2.39	1.22–4.71	0.011	2.38	1.60–3.56	<0.001
Tonga	0.70	0.52–0.94	0.017	0.95	0.75–1.21	0.692	2.47	1.77–3.45	<0.001	1.16	0.77–1.76	0.470	3.34	1.55–7.19	0.002	1.84	1.41–2.41	<0.001
Vanuatu	0.69	0.40–1.19	0.180	1.78	1.14–2.78	0.011	1.33	0.64–2.75	0.447	1.51	0.68–3.34	0.307	1.45	0.37–5.66	0.591	1.93	1.26–2.94	0.002
Pooled	0.78	0.68–0.91	0.001	1.40	1.12–1.75	0.004	1.78	1.53–2.06	<0.001	1.57	1.33–1.85	<0.001	1.72	1.33–2.23	<0.001	1.72	1.51–1.95	<0.001

OR = odds ratios; 95% CI = 95% confidence interval; multivariate logistic models adjusted for the other independent variables.

**Table 5:** Joint influence of risk behaviors (smoking, drinking, drug use and missing school) on physical fighting in young adolescents in 6 Western Pacific countries.

Country	Number of Risk Behaviors									
	0	1			2			≥3		
		OR	95% CI	*p*	OR	95% CI	*p*	OR	95% CI	*p*
Cook Islands	Referent	1.89	1.29–2.76	0.001	4.34	2.73–6.90	<0.001	4.09	2.35–7.12	<0.001
Kiribati	Referent	1.61	1.20–2.17	0.002	2.59	1.88–3.58	<0.001	3.53	2.28–5.46	<0.001
Samoa	Referent	2.00	1.41–2.85	<0.001	3.08	1.77–5.36	<0.001	5.91	3.93–8.90	<0.001
Solomon	Referent	2.24	1.51–3.34	<0.001	2.69	1.38–5.25	0.004	5.90	3.59–9.70	<0.001
Tonga	Referent	2.24	1.74–2.88	<0.001	4.96	3.62–6.79	<0.001	6.21	3.78–10.22	<0.001
Vanuatu	Referent	2.06	1.20–3.51	0.008	2.17	0.76–6.14	0.146	3.82	1.53–9.55	0.004
Pooled	Referent	1.99	1.73–2.29	<0.001	3.52	2.94–4.21	<0.001	4.95	4.03–6.07	<0.001

OR = odds ratios; 95% CI = 95% confidence interval; multivariate logistic models adjusted for age and sex.
